# TALAIA: a 3D visual dictionary for protein structures

**DOI:** 10.1093/bioinformatics/btad476

**Published:** 2023-08-07

**Authors:** Mercè Alemany-Chavarria, Jaime Rodríguez-Guerra, Jean-Didier Maréchal

**Affiliations:** Insilichem, Department of chemistry, Universitat Autònoma de Barcelona, Bellaterra 08193, Barcelona, Spain; Insilichem, Department of chemistry, Universitat Autònoma de Barcelona, Bellaterra 08193, Barcelona, Spain; Insilichem, Department of chemistry, Universitat Autònoma de Barcelona, Bellaterra 08193, Barcelona, Spain

## Abstract

**Motivation:**

Graphical analysis of the molecular structure of proteins can be very complex. Full-atom representations retain most geometric information but are generally crowded, and key structural patterns can be challenging to identify. Non-full-atom representations could be more instructive on physicochemical aspects but be insufficiently detailed regarding shapes (e.g. entity beans-like models in coarse grain approaches) or simple properties of amino acids (e.g. representation of superficial electrostatic properties). In this work, we present TALAIA a visual dictionary that aims to provide another layer of structural representations.

TALAIA offers a visual grammar that combines simple representations of amino acids while retaining their general geometry and physicochemical properties. It uses unique objects, with differentiated shapes and colors to represent amino acids. It makes easier to spot crucial molecular information, including patches of amino acids or key interactions between side chains. Most conventions used in TALAIA are standard in chemistry and biochemistry, so experimentalists and modelers can rapidly grasp the meaning of any TALAIA depiction.

**Results:**

We propose TALAIA as a tool that renders protein structures and encodes structure and physicochemical aspects as a simple visual grammar. The approach is fast, highly informative, and intuitive, allowing the identification of possible interactions, hydrophobic patches, and other characteristic structural features at first glance. The first implementation of TALAIA can be found at https://github.com/insilichem/talaia.

## Graphical Abstract

**Figure:**
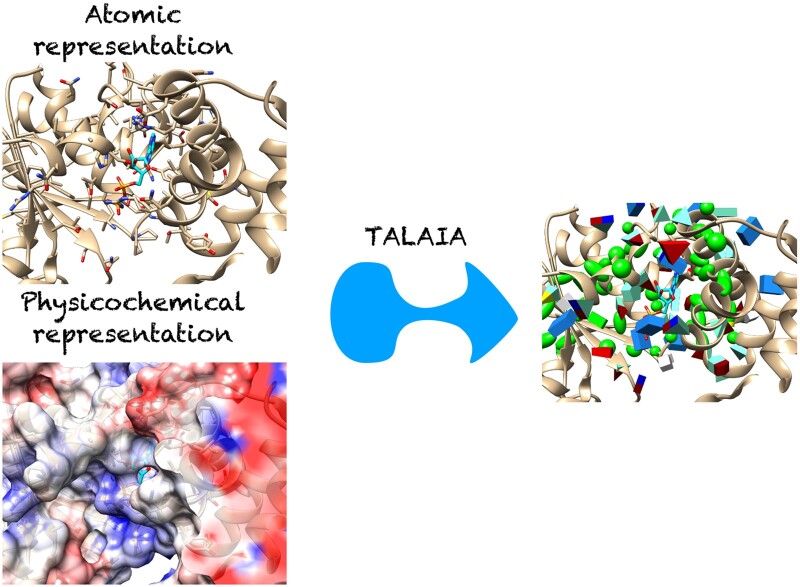
TALAIA is a 3D visual dictionary that simplifies the representation of amino acids while keeping their overall shape and relevant physicochemical properties.

## 1 Introduction

Molecular sciences are intrinsically related to our understanding of the three-dimensional (3D) nature of molecules. Graphic representations, like those provided by computer software, allow us to perceive, describe, analyze, demonstrate, and communicate molecular knowledge, including morphological, dynamic properties, and reactivity. These representations stand on a series of conventions and simplifications that relate to forms (e.g. spheres for atoms, cylinders for bonds, etc.), colors (yellow for sulfur, red for oxygen, etc.), or textures (e.g. dot lines for weak interactions or depth of a bond for simple, double, or triple).

Full-atom models are frequently challenging to interpret large systems like proteins, while non-full-atom representations can hide crucial in-depth information. For example, the ribbon representation of the backbone may obscure interactions of the peptide chain’s oxygen and/or nitrogen atoms. Physicochemical maps, like electrostatic ones, could be highly informative but miss the atomic details of the systems. A frequent solution consists of allying both full-atom models with physicochemical maps. However, those hybrid representations could rapidly lead to cluttered modeling and blurred visualization. Graphic representations that combine morphological (meaning maintaining most of the shape of the amino acids) and physicochemical information are primarily absent from structural biology to date. The unique exception may be the Symbol Nomenclature for Glycans (Varki *et al.* 2015), a visual code proposed for saccharides. To some extent, the pipes-and-planks models of DNA or RNA embody this concept by representing backbone and nitrogen bases in an intermediate graphical manner (Couch *et al.* 2006). Similarly, pharmacophores maintain the key structural 2D or 3D features of small molecules by blurring their complete atomic nature but maintain their fundamental physicochemical features considering, eventually, in front of their closest environment (Schaller *et al.* 2020).

Here, we propose TALAIA as a visual dictionary for proteins. TALAIA provides a visual representation of amino acid side chains that merges structural and some physicochemical information and could serve in inspecting protein structures, evaluating interactions, or understanding the driving forces of dynamical processes (e.g. a folding process). TALAIA can be applied to static structures and ensembles like trajectories obtained from molecular dynamics simulations.

## 2 Availability and implementation

The first version of TALAIA is available as a UCSF Chimera (Pettersen *et al.* 2004) extension and can be downloaded from the GitHub repository at https://github.com/insilichem/talaia (see repository to install). Expansion toward other platforms is underway.

**Figure 1.**
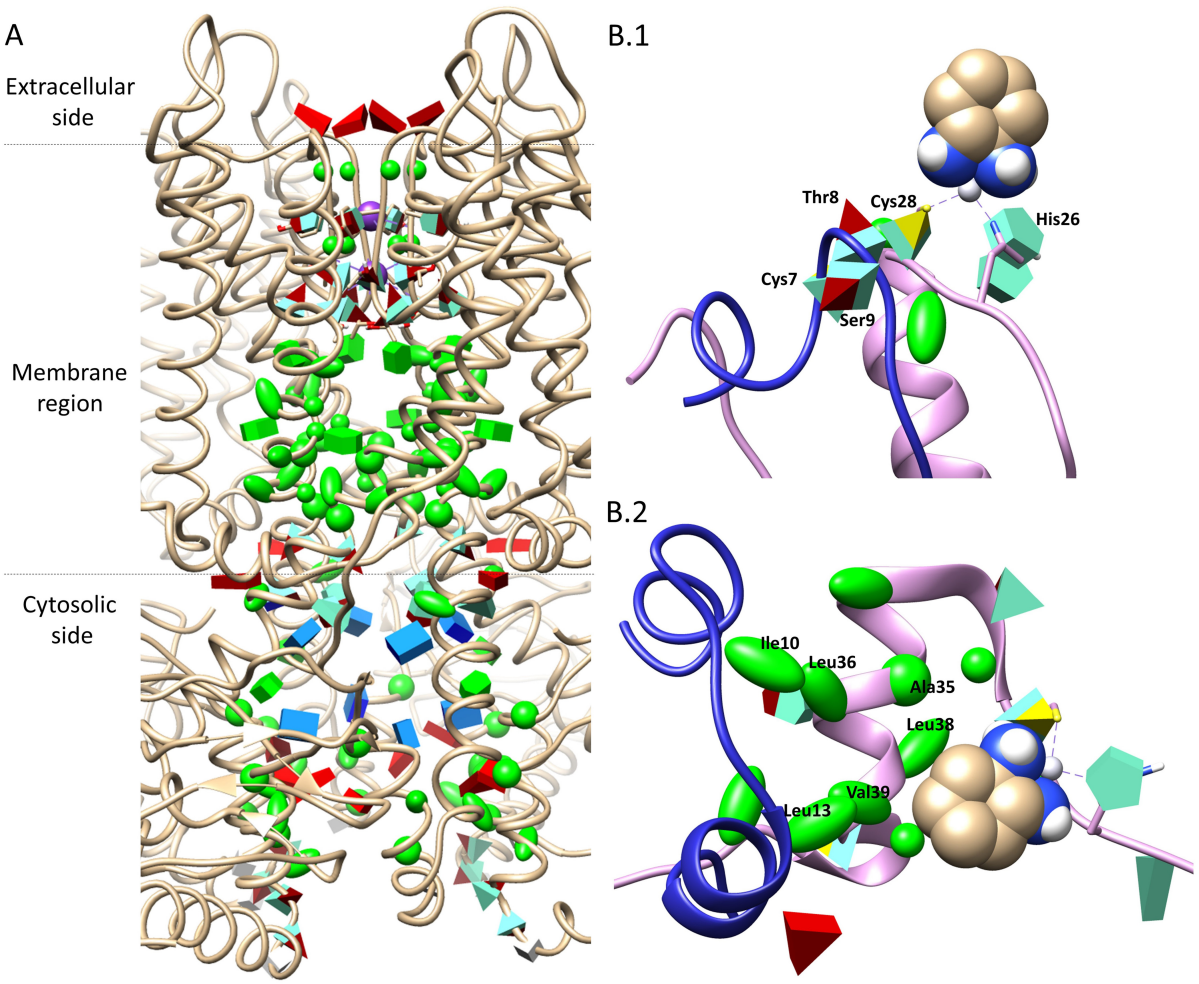
(A) cAMP-bound potassium channel MloK1 (PDB: 6eo1). Residues inside the channel are represented with TALAIA. (B) Snapshots from the molecular dynamics’ simulation of the insulin–oxaliplatin complex with TALAIA’s representation for residues in 8 Å range from the drug, showing initial conformation (B.1) and final conformation (B.2).

In brief, the program reads the identity of the residues and the atomic coordinates from the input file of the protein structure. Then it displays the corresponding graphical object on top of Chimera’s representation. These objects can overlap easily with the representation of the system’s secondary structure and individual atoms (Fig. 1). This can be helped by setting the transparency of the figures manually.

## 3 Description and illustrative cases

### 3.1 Dictionary overview

TALAIA classifies amino acids into five families: hydrophobic, neutral polar residues, positively and negatively charged residues, and proline (Table 1). Most of TALAIA’s objects are based on conventions that are standards for molecular scientists. Regarding colors, hydrophobic residues are depicted in green, positively charged groups in dark blue, and negatively charged in red. Regarding forms, they tend to reproduce the most critical aspects of the geometry of the amino acids side chains. Details are provided below.

**Table 1.** Visual dictionary classification.

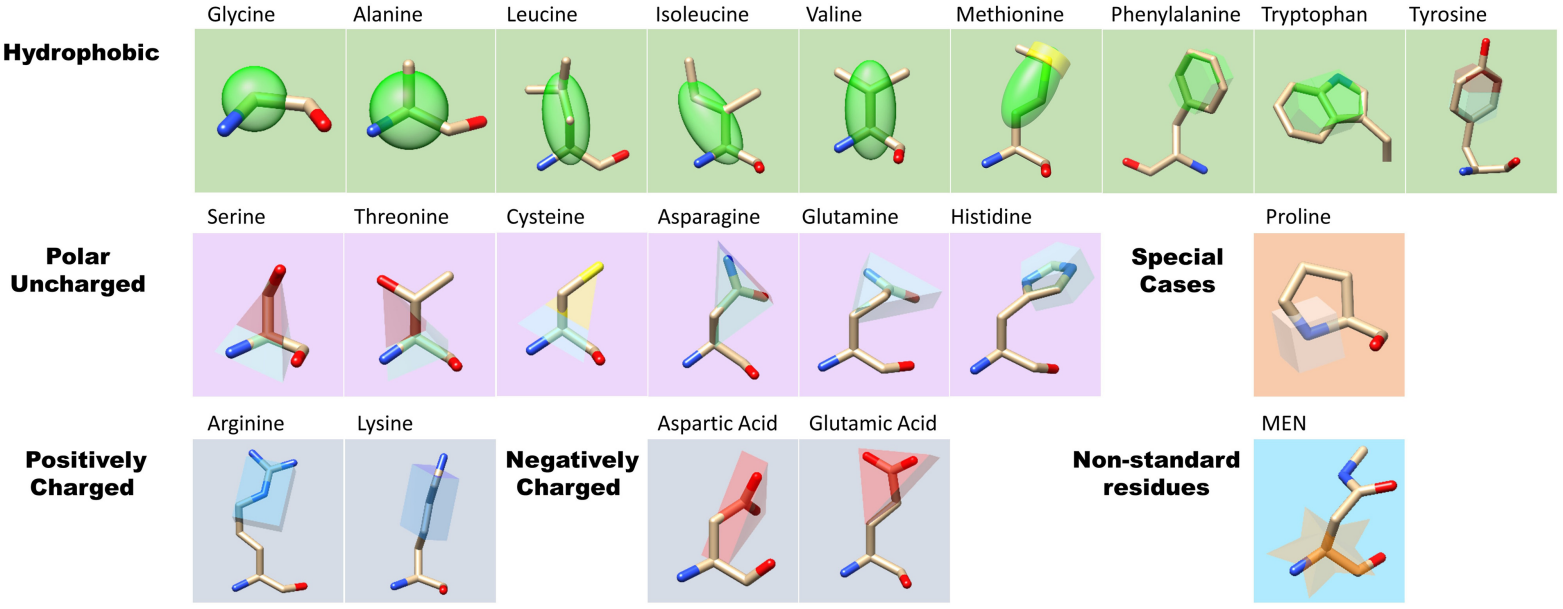

Aliphatic groups are presented in spheric (Glycine, Alanine), ellipsoid (Valine, Leucine, Isoleucine, Methionine), or hexagonal (Phenylalanine, Tryptophan) shapes. The elongation of the ellipsoid and hexagons are functions of the dimensions of the side chain and take place along the central axis of the amino acids. Methionine has a yellow ring around the ellipsoid’s end where the sulfur atom is located. Neutral polar side chains, Serine, Threonine, and Cysteine are represented by a pyramid to indicate the direction of the polarity. The vertex points to the polar group, and the center is on the Cα. The pyramid tip is yellow for Cys (the thiol group) and red for Ser and Thr (the hydroxyl group). Triangular prisms represent Glutamine and Asparagine. The prism’s base is colored dark blue and red and located over the polar atoms of the amide group. Tyrosine is represented by a hexagon that overlaps with the residue’s ring, and the hydroxyl moiety is colored red.

Histidine is depicted by an aquamarine pentagon overlapping the residue’s ring when the residue is found in the neutral state. The color of the entire figure can change according to its protonation state, dark blue when fully protonated and red when fully deprotonated.

Positively charged residues Lysine and Arginine are represented by a blue rectangular box. The side of the cuboid where the amino group (Lys) and the guanidyl group (Arg) lie is colored in a darker shade of blue. Aspartate and Glutamate side chains are depicted with the exact figure as their amide forms, Asparagine and Glutamine, and where the carboxylic group is found is depicted in a darker shade of red. Proline is a particular case since its structural characteristics and physicochemical properties can’t be included in any previous groups. Proline is therefore provided with a unique code as a small gray cube centered on the Cα. This shape is chosen due to the residue’s structural role. Along the same line, an orange star placed on top of the Cα was attributed to non-standard amino acids. Specific objects for small molecules (ligands, solvents, or ions) in TALAIA are not considered yet.

### 3.2 Illustrative cases

For the sake of presenting TALAIA to the community, we selected two cases for which a reduced number of static pictures are already informative. We selected the potassium channel of Mesorhizobium japonicum with PDB code 6eo1 (Kowal *et al.* 2018) and two snapshots from the conformational transition of the oxaliplatin once bound to insulin obtained by molecular dynamics (Sciortino *et al.* 2019).

Regarding the potassium channel (Fig. 1A), TALAIA allows us to identify subregions immediately: (1) a patch of negatively charged residues at the extracellular entrance of the pore, (2) small residues and neutral polar residues at the core (the selectivity filter), and (3) a hydrophobic region formed mainly by S6 and C-linker at the end of the transmembrane region. This representation allows fast recognition of a ring of positively charged arginine residues where the cyclic nucleotide-binding domain binds near the membrane interface. Below that region, negatively charged residues are located and mark the pore’s cytosolic end.

Regarding the insulin–oxaliplatin system, Fig. 1B presents two snapshots of the conformational transition observed in a molecular dynamics simulation for one of the low-energy complexes obtained by protein-ligand dockings (Sciortino *et al.* 2019). This complex results from the cleavage of the Cys28–Cys7 disulfide bridge so that Cys28 can coordinate the platinum ion with His26. TALAIA depiction allows us to rapidly highlight that during the transition, the second coordination sphere interaction of the metal with Thr8 and Ser9 is lost (Fig. 1B.1). This allows the NTer part of the helix to unfold, and finally, the aromatic part of the drug can reach a hydrophobic core formed by Ile10, Leu13, Ala35, Leu36, Leu38, and Val39 (Fig. 1B.2).

To conclude, we believe that TALAIA provides an interesting complementary tool for the molecular inspection of biomolecular systems by affording a visual code that preserves global geometric blueprints of amino acids that are physicochemically sound.

## Data Availability

The data underlying this article (code, installation procedure and tutorials) are available in https://github.com/insilichem/talaia. Guidance steps for illustrative cases including those produced in this manuscript are also available.
